# Segmentation of retinal microaneurysms in fluorescein fundus angiography images by a novel three-step model

**DOI:** 10.3389/fmed.2024.1372091

**Published:** 2024-06-19

**Authors:** Jing Li, Qian Ma, Mudi Yao, Qin Jiang, Zhenhua Wang, Biao Yan

**Affiliations:** ^1^Eye Institute and Department of Ophthalmology, Eye and ENT Hospital, State Key Laboratory of Medical Neurobiology, Fudan University, Shanghai, China; ^2^College of Information Science, Shanghai Ocean University, Shanghai, China; ^3^Department of Ophthalmology, General Hospital of Ningxia Medical University, Ningxia, China; ^4^Department of Ophthalmology and Optometry, The Affiliated Eye Hospital, Nanjing Medical University, Nanjing, China; ^5^Department of Ophthalmology, Shanghai General Hospital, Shanghai Jiao Tong University School of Medicine, Shanghai, China

**Keywords:** diabetic retinopathy, segmentation model, microaneurysms, fluorescein fundus angiography, computer-aided diagnosis

## Abstract

**Introduction:**

Microaneurysms serve as early signs of diabetic retinopathy, and their accurate detection is critical for effective treatment. Due to their low contrast and similarity to retinal vessels, distinguishing microaneurysms from background noise and retinal vessels in fluorescein fundus angiography (FFA) images poses a significant challenge.

**Methods:**

We present a model for automatic detection of microaneurysms. FFA images were pre-processed using Top-hat transformation, Gray-stretching, and Gaussian filter techniques to eliminate noise. The candidate microaneurysms were coarsely segmented using an improved matched filter algorithm. Real microaneurysms were segmented by a morphological strategy. To evaluate the segmentation performance, our proposed model was compared against other models, including Otsu's method, Region Growing, Global Threshold, Matched Filter, Fuzzy c-means, and K-means, using both self-constructed and publicly available datasets. Performance metrics such as accuracy, sensitivity, specificity, positive predictive value, and intersection-over-union were calculated.

**Results:**

The proposed model outperforms other models in terms of accuracy, sensitivity, specificity, positive predictive value, and intersection-over-union. The segmentation results obtained with our model closely align with benchmark standard. Our model demonstrates significant advantages for microaneurysm segmentation in FFA images and holds promise for clinical application in the diagnosis of diabetic retinopathy.

**Conclusion:**

The proposed model offers a robust and accurate approach to microaneurysm detection, outperforming existing methods and demonstrating potential for clinical application in the effective treatment of diabetic retinopathy.

## 1 Introduction

Diabetic retinopathy (DR) is known as a blinding eye disease in the working population. Most of the patients with type 1 diabetes mellitus and nearly 60% of the patients with type 2 diabetes mellitus will develop retinopathy following a long duration of diabetes (≥20 years). However, it is difficult to detect DR until it develops into the advanced vision-threatening stage (1). DR is often divided into two stages: non-proliferative DR (NPDR) and proliferative DR (PDR). In the NPDR stage, hyperglycemia can cause serious injuries to retinal capillaries, which can weaken the capillary walls and lead to the occurrence of microaneurysms (MAs). MAs are the small outpouchings of retinal capillaries and the early signs of NPDR, as well as the indicators for DR progression (2, 3). MAs appear as small, reddish, and circular shapes in color fundus images. They can be clinically identified by ophthalmoscopy as the deep-red dots varying from 10 to 100 μm in diameter (4, 5). Thus, automatic detection of MAs is important for DR diagnosis, which can help in controlling and retarding visual loss.

Previous studies have reported that several imaging modalities have been developed for MA detection, including color fundus images (6), optical coherence tomography angiography (OCTA) (7), and fluorescein fundus angiography (FFA) (8). Colored fundus photography has often been used due to its low cost compared with Optical coherence tomography machines. Walter et al. proposed a method for the automatic detection of MAs based on diameter closure and kernel density estimation (6). Melo et al. proposed a method for MA detection using the sliding band filter algorithm in color fundus images (9). MAs are situated on retinal capillaries and are not often visible, which makes them difficult to distinguish from the noises and pigmentation variations in color fundus images. An OCTA can provide detailed visualization of vascular perfusions. However, optical coherence tomography (OCT) machines are very expensive, and the interpretation of OCTA data is still challenging due to the complicated image artifacts and elusive algorithmic details of OCTA data (10, 11). FFA can be used for the detection of small changes in retinal vessels. The small and leaky MAs are easily ignored without the aid of FFA. FFA is highly effective in detecting MAs, especially when MAs are close to the vessels or too small to distinguish (12, 13). However, objective segmentation of MAs in FFA images is still challenging because MA segmentation requires laborious manual segmentation by experienced graders. Therefore, it is necessary to develop a model for automatic detection of MAs in FFA images for DR diagnosis.

Computer-assisted MA detection is important for DR diagnosis. Baudoin et al. used a mathematical morphology method to remove vessels and applied a top-hat transformation with the linear structuring elements to detect MAs (14). Spencer et al. proposed an image correction procedure for MA segmentation by calculating the true- and false-positive rates (15). Mendonca et al. further improved this method by altering the pre-filtering and classification procedures. However, shade corrections may produce false positives caused by the darkening of regions close to the bright patterns (16). Walter applied mathematical morphology to segment the vascular trees of retinal angiograms. This algorithm can extract patterns if vein width is constant, but it cannot extract them from narrower/wider veins (17). Zhang et al. proposed a model based on the dynamic thresholding and correlation coefficients of a multi-scale Gaussian template (18). Antal and Hajdu proposed an ensemble-based method for MA detection by selecting an optimal combination of pre-processing methods and candidate extractors (19). Saleh et al. developed a DR detection system based on the Gaussian filter, a multi-layered dark object filtering method, and a singular spectrum analysis (20). Despite their clinical significance, MAs pose challenges for accurate detection due to their low-contrast and close resemblance to blood vessels. Thus, further study is necessary to refine MA detection algorithms and enhance accuracy, particularly in FFA images. In this study, we present a novel model for the automatic detection of MA lesions in FFA images. Our proposed model comprises pre-processing of FFA images, followed by coarse segmentation of candidate MA regions and fine segmentation of MA regions. Subsequently, comparative studies were conducted to assess the MA detection performance of the proposed model.

## 2 Materials and methods

### 2.1 The proposed model for MA detection

**Figure 1:**
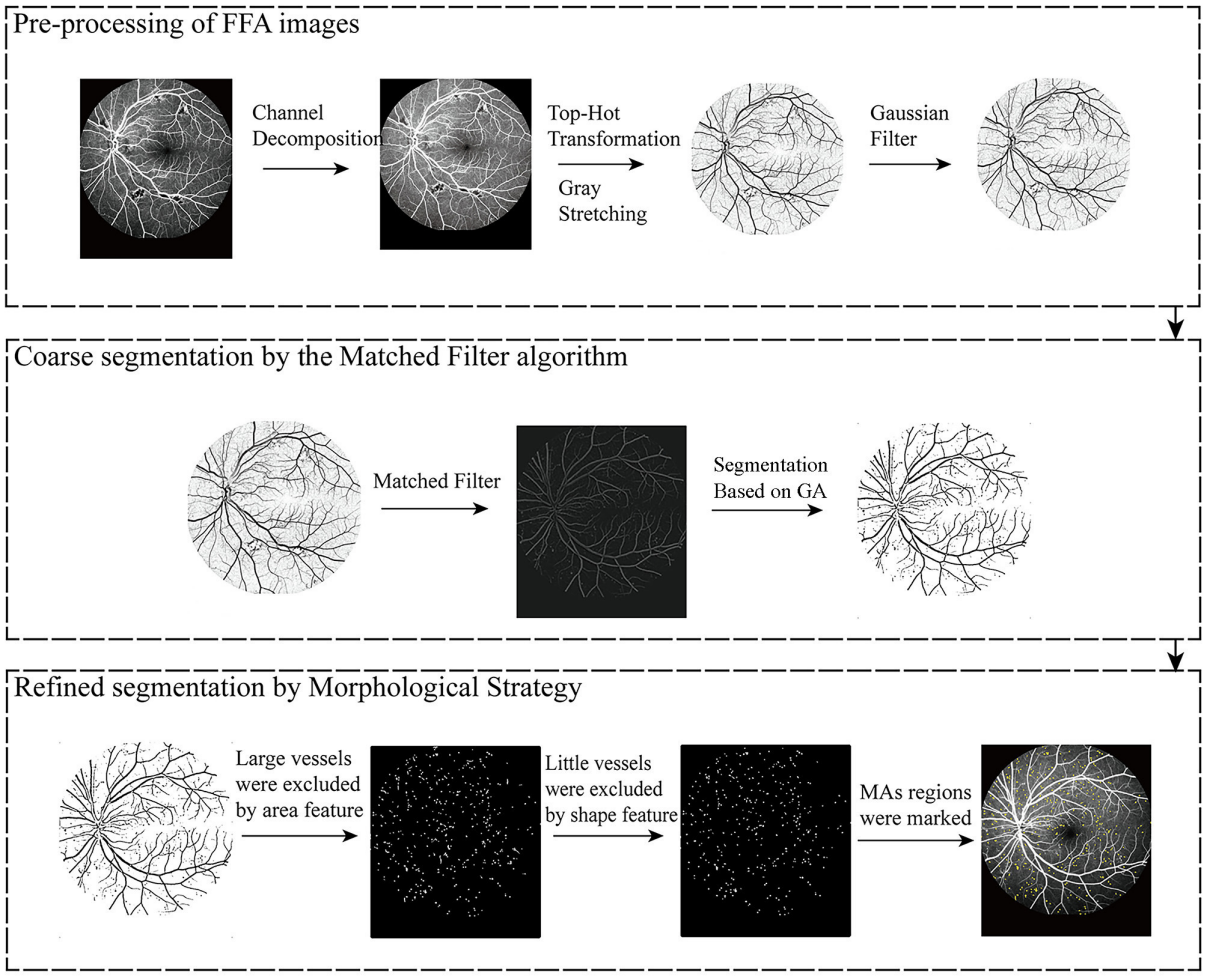
Flow chart of the proposed model for microaneurysm (MA) segmentation.

The flowchart of the proposed MA detection model is shown in Figure 1, including pre-processing of FFA images, coarse segmentation of candidate MA regions by the matched filter (MF) algorithm, and fine segmentation of MA regions by the morphological strategy.

### 2.2 Pre-processing of FFA images

High-noise and low-contrast can pose great difficulties for the identification of MAs in FFA images. In the pre-processing step, the FFA images underwent decomposition into individual channels to alleviate computational demands, given that the pixel values across each channel were identical. Subsequently, each single channel underwent processing, employing top-hat transformation and gray-stretching (21) to enhance the contrasts between MAs and the background. Following this processing, the processed result underwent further refinement via a Gaussian filter to reduce noise.

The top-hat transformation was defined according to Equation (1) (22):


(1) $$\begin{array}{l} I_{th}\left(x,y\right)=I\left(x,y\right)-I\left(x,y\right)\circ B\left(u,v\right) \end{array}$$

where *I*(*x, y*) refers to the grayscale image, *B*(*u, v*) refers to the structural element constructed as a circle with a radius of 45 pixels, and ° refers to the open operation. Opening of *I*(*x, y*) by *B*(*u, v*) was defined according to Equation (2):


(2) $$\begin{array}{l} I\left(x,y\right)\circ B\left(u,v\right)=\left(I\left(x,y\right)\Theta B\left(u,v\right)\right)⊕B\left(u,v\right) \end{array}$$

where Θ and ⊕ refer to the erosion and dilation operations, respectively. The erosion and dilation of *I*(*x, y*) by *B*(*u, v*) were defined according to Equations (3) and (4):


(3) $$\begin{array}{l} I\left(x,y\right)\Theta B\left(u,v\right)=\underset{u,v}{\min}(I(x+u,y+v)-B(u,v)) \end{array}$$

(4) $$\begin{array}{l} I\left(x,y\right)⊕B\left(u,v\right)=\underset{u,v}{\min}(I\left(x-u,y-v\right)+B\left(u,v\right)) \end{array}$$

Gray-stretching was defined according to Equation (5) (23):


(5) $$\begin{array}{l} I_{new}=\left(\frac{G_{\max}-G_{\min}}{I_{\max}-I_{\min}}\right)\left(I-I_{\min}\right)+G_{\min} \end{array}$$

where *I*_max_ and *I*_min_ refer to the largest and smallest gray values in the original images, respectively. *G*_max_ and *G*_min_ refer to the largest and smallest gray values in the transformed images.

The Gaussian filter was defined according to Equation (6) (24):


(6) $$\begin{array}{l} G\left(x,y\right)=\frac{1}{\sqrt{2\pi \sigma }}e^{\frac{-\left(x^{2}+y^{2}\right)}{2\sigma^{2}}} \end{array}$$

where σ^2^ refers to the variance of the Gaussian filter.

In the pre-processing step, top-hat transformation, gray-stretching, and a Gaussian filter were employed for MA extraction by strengthening, enhancing, and denoising. A top-hat transformation was used to highlight the object edges and remove distracting information such as background noises. Gray-stretching mapped the grayscale ranges of FFA images. The Gaussian filter smoothed FFA images and removed irregular details such as noise points and burrs in the FFA images.

### 2.3 Coarse segmentation of MAs by the MF algorithm

The candidate MA regions in the FFA images were detected using the MF algorithm. MF was initially proposed by Chaudhuri et al. (25) for blood vessel extraction. Analogous to the matching filter concept in signal processing, a blood vessel image can be interpreted as a signal. Blood vessels exhibit characteristics such as a narrow range of width variation and parallel inner walls. Based on the prior knowledge, MF can construct a template to match the cross-sectional structure of blood vessels. Consequently, when the blood vessel component is input, a higher value is yielded, whereas a lower value is produced for the background, facilitating the separation of blood vessels. Hence, MF effectively enhances blood vessels and suppresses background noises.

MF was defined according to Equation (7) (26):


(7) $$\begin{array}{l} f\left(x,y\right)=\frac{1}{\sqrt{2\pi s^{2}}}e^{\frac{-x^{2}}{2s^{2}}}-m,\;|x|\leq t\times s,|y|\leq \frac{L}{2} \end{array}$$

where *s* refers to the filter scale, *m* is used for normalizing the mean value of the filter to 0, which is defined as Equation (8), *L* refers to the neighborhood length along the *y*-axis and is used to smooth the noises. *L* was deduced by *s*. When *s* was small, *L* was set relatively small, and vice versa. The criterion *t* is a constant and was set to 3 (27).


(8) $$\begin{array}{l} m=\frac{\int_{-ts}^{ts}\frac{1}{\sqrt{2\pi s^{2}}}e^{\frac{-x^{2}}{2s^{2}}}dx}{2ts} \end{array}$$

The performance of the MF algorithm is heavily reliant on the design of the template. Poorly designed templates or significant deviations from the actual blood vessel structure can result in inaccurate extraction or an abundance of noise. Genetic algorithms (GA), an optimization technique introduced by John Holland, offer a solution to this challenge. GA mimics natural selection and genetic mechanisms to search for optimal solutions within the solution space. By using GA, one can efficiently explore and identify template configurations that yield improved accuracy and robustness in vessel extraction.

Hence, GA can be utilized to automatically adjust the threshold value of MF to accommodate the morphological features of blood vessels in various images. The GA process comprises five key steps: population initialization, fitness assessment, selection, crossover, and mutation. In the population initialization step, chromosome length was set to 8 and population size was set to 10. In the fitness assessment step, the efficacy of a solution was determined using a fitness function, where solutions with higher fitness were deemed superior. In our study, the fitness function of the GA is defined as in Equation (9) (28). In the selection step, the elitism strategy was adopted. In the crossover step, the crossover probability was set to 0.7. In the mutation step, the mutation probability was set to 0.4. In the later stages of the genetic algorithm's evolution, adjustments were made to both the crossover and mutation probabilities, setting them to 0.3 each.

Through iterative optimization via GA, the MF template that most accurately aligns with blood vessels can be gradually identified, enabling the identification of all candidate MAs.


(9) $$\begin{array}{l} f=p_{1}\times p_{2}\times {\left(\mu_{1}-\mu_{2}\right)}^{2} \end{array}$$

where *p*_1_ and *p*_2_ refer to the number of the target pixels and background pixels, respectively, μ_1_ and μ_2_ refer to the average gray values of the target pixels and background pixels, respectively. *f* is the fitness value.

### 2.4 Fine segmentation of MAs by the morphological strategy

Real MA regions were determined by the morphological strategy, including removing vessels, hemorrhages, and exudates from the candidate MA regions based on area features and shape features, respectively. Previous studies have developed multiple image processing and machine learning algorithms for the automatic detection of MAs and recognized that the area size of MAs was typically between 5 and 100 pixels. In addition, real MAs were often localized next to the capillaries, appearing as dotted or rounded structures (29–31). The vessels, hemorrhages, and exudates were removed from the candidate MA regions according to Equation (10). Hemorrhages and exudates caused by the injured vessels were removed from the candidate MA regions according to Equation (11) and the threshold for roundness was set to 0.51.


(10) $$\begin{array}{l} I\left(x,y\right)=\{\begin{array}{c} 0,\;S>100\\ 1,\;5\leq S\leq 100\\ 0,\;S<5 \end{array} \end{array}$$

(11) $$\begin{array}{l} Roundess=\frac{4\pi S}{C^{2}} \end{array}$$

where *S* refers to the pixels of the candidate MA regions and *C* refers to the circumference of the contour.

### 2.5 Dataset

**Figure 2:**
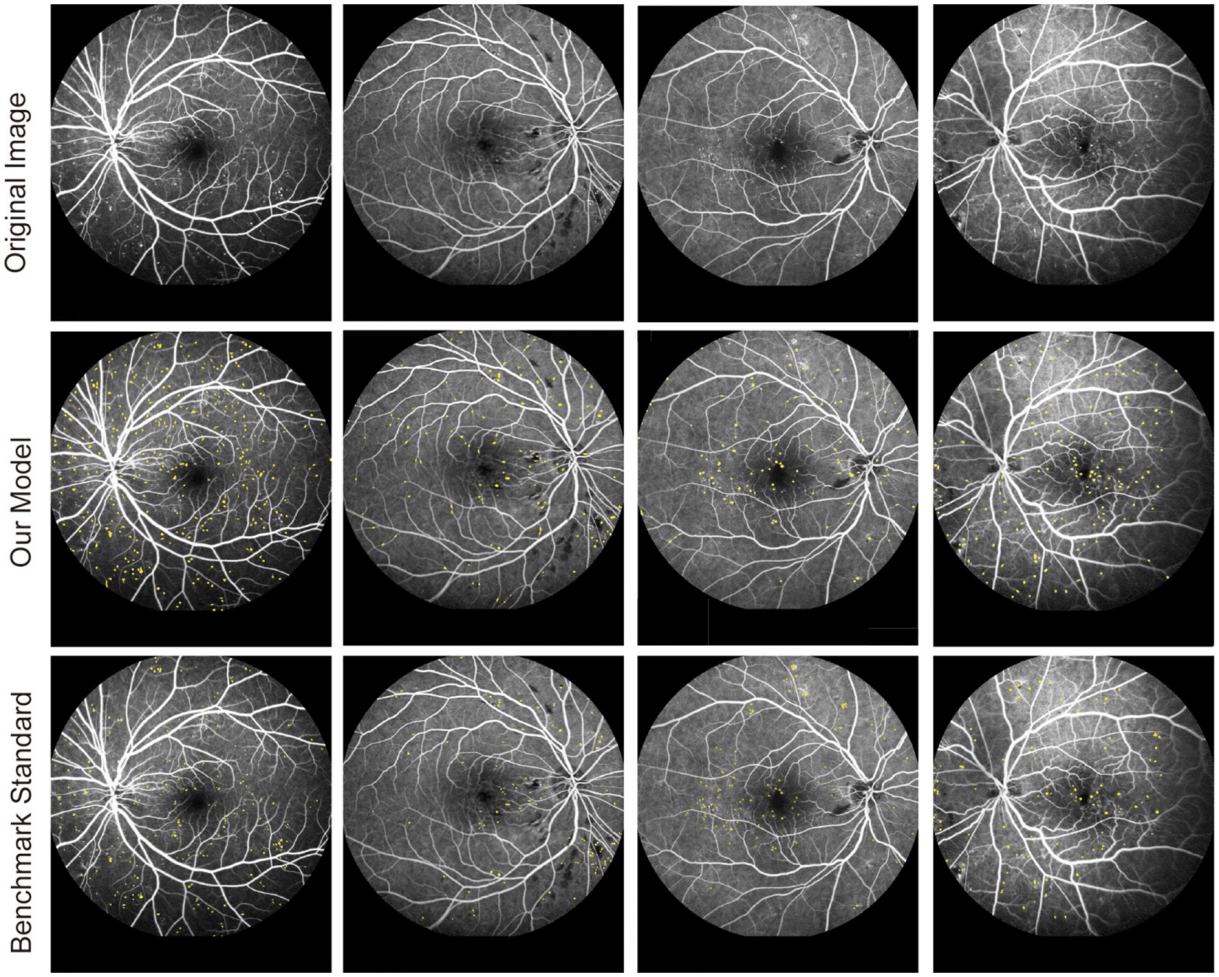
Original fluorescein fundus angiography (FFA) images and segmentation results of MAs by the proposed model and retinal clinicians.

The FFA dataset comprises 1,010 FFA images, each with dimensions of 768 × 868 pixels, obtained from 65 eyes of 60 DR patients aged between 31 and 81 years. These patients underwent FFA examinations at the Eye Hospital affiliated with Nanjing Medical University between 2015 and 2019. The FFA images were captured using Heidelberg Retina Angiography (Heidelberg Engineering, Germany) by experienced clinicians. Notably, the FFA dataset did not include blurry or overexposed images. For labeling MAs in FFA images, three retinal clinicians with over 10 years of experience independently annotated MAs, serving as the benchmark standard. Patients with FFAs indicating mild or moderate DR were eligible for inclusion. The following exclusion criteria were used: (1) presence of other ocular diseases unrelated to diabetes, such as retinal arteriovenous obstruction, age-related macular degeneration, glaucoma, and uveitis; (2) any condition causing poor image quality or inability to visualize the optic disc and vessels, such as dense cataracts or corneal opacity; and (3) history of previous ophthalmological interventions, such as laser photocoagulation, vitrectomy, or anti-vascular endothelial growth factor injection. To ensure the reliability and validity of segmentation results, FFA images were independently divided into three sets: 830 images for training, 90 images for testing, and 90 images for validation. Figure 2 shows the original FFA images and MA detection results by the proposed model and benchmark standard.

Another publicly available dataset was utilized to assess the performance of MA detection. This dataset consisted of FFA images obtained from diabetic patients. The images were captured as part of a study conducted at the Persian Eye Clinic (Feiz Hospital), affiliated with the Isfahan University of Medical Sciences. The dataset comprised retinal images from 70 patients, with 30 samples categorized as normal and 40 samples representing various stages of DR.

### 2.6 Evaluation metrics

Five different metrics, including accuracy (Acc) (30), sensitivity (Se) (30), specificity (Sp) (30), positive predictive value (PPV) (31), and intersection-over-union (IOU) (32), were employed to evaluate the detection performance of MAs according to Equations (12–16):


(12) $$\begin{array}{l} Acc=\frac{TP+TN}{TP+FP+TN+FN} \end{array}$$

(13) $$\begin{array}{l} Se=\frac{TP}{TP+FN} \end{array}$$

(14) $$\begin{array}{l} Sp=\frac{TN}{TN+FP} \end{array}$$

(15) $$\begin{array}{l} PPV=\frac{TP}{TP+FP} \end{array}$$

(16) $$\begin{array}{l} IOU\;=\;\frac{TP}{TP\;+\;FP\;+\;FN} \end{array}$$

where TP denotes the region that was predicted as MAs and was real MAs; FP denotes the region that was predicted as MAs but was background; TN denotes the region that was predicted as background and was real background; and FN denotes the region that was predicted as background but was MAs. Accuracy (Acc) is defined as the measure providing the ratio of total well-segmented pixels based on the gold standard for hand-labeled detection. Sensitivity (Se) and specificity (Sp) measures the ability of the model to detect well-segmented MAs and background pixels, respectively. PPV represents the correct proportion of the sample with a positive prediction. The IOU reflects the degree of coincidence between the MA detection result of the proposed model and the benchmark standard.

### 2.7 Implementation

All experiments were conducted on a PC with an Intel Core processor running at 2.50 GHz and equipped with 8 GB of RAM, using the MATLAB 2013a software.

## 3 Results

Our proposed model encompassed the pre-processing of FFA images, followed by coarse segmentation of candidate MA regions and fine segmentation of MA regions. To assess the MA detection performance of our proposed model, two distinct experiments were conducted. In Experiment 1, our proposed model was juxtaposed against the MF model optimized by the GA algorithm (referred to as MAs-MF). In Experiment 2, our proposed model was compared against previous MA detection models. To maintain the integrity of our experiments, the outcomes presented for the clinician in Table 1 were segmented by a skilled clinician who did not participate in the dataset labeling process.

**Table 1:** Performance comparison between our proposed model and the microaneurysms-matched filter (MAs-MF) model.

**Model**	**Evaluation metrics**				
	**Acc (%)**	**Se (%)**	**Sp (%)**	**PPV (%)**	**IOU (%)**
Clinician	99.94 ± 0.04	96.65 ± 0.08	99.96 ± 0.02	92.91 ± 0.09	90.02 ± 0.08
MAs-MF	99.43 ± 0.06	90.95 ± 0.46	99.46 ± 0.05	42.42 ± 0.97	40.64 ± 1.07
Our model	99.80 ± 0.05	92.10 ± 0.20	99.85 ± 0.04	75.07 ± 0.44	70.57 ± 0.55

### 3.1 The ablation experiment suggests that our proposed model improves MA detection performance

**Figure 3:**
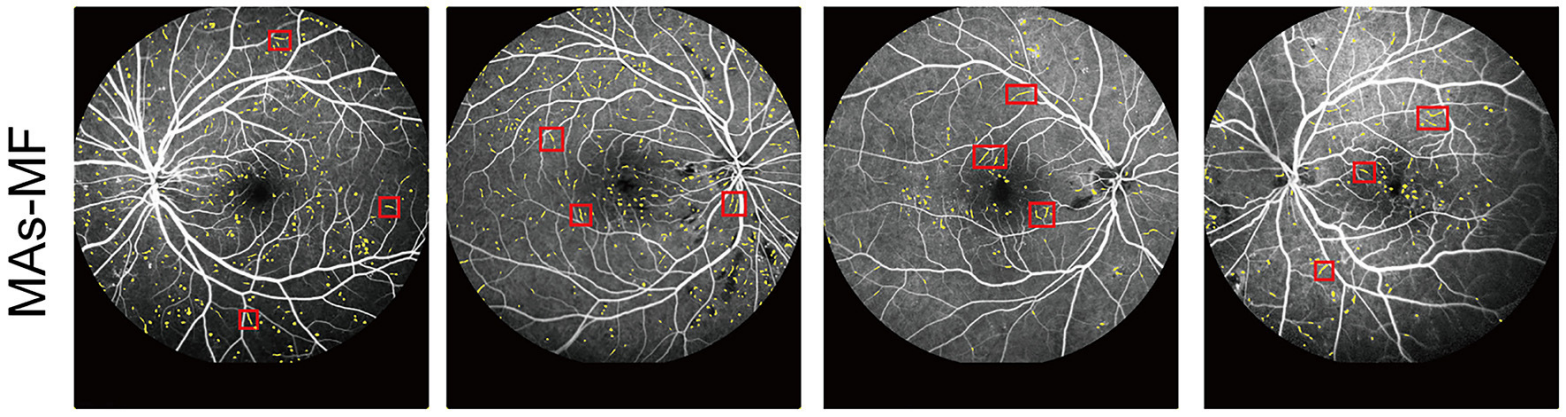
Detection results of MAs by microaneurysms-matched filter (MAs-MF).

We compared our proposed model against the MAs-MF model to evaluate MA detection performance. The results of MA detection are shown in Figure 3. The metrics of MA detection are shown in Table 1.

From Figure 3 and Table 1, we can observe that there were several label errors of small blood vessels for MA detection results in the MAs-MF model, as shown in the red squares in Figure 3. Compared with the MAs-MF model, the MA detection performance of the proposed model was close to the MA detection results of the clinicians. Compared with the MAs-MF model, the proposed model had greater values of accuracy (Acc), sensitivity (Se), specificity (Sp), PPV, and IOU, which were 99.80 (0.37↑), 92.10 (1.15↑), 99.85 (0.39↑), 75.07 (32.65↑), and 75.57 (29.93↑), respectively.

### 3.2 The comparison experiment suggests that the proposed model has an obvious MA detection advantage over previous MA detection models

**Figure 4:**
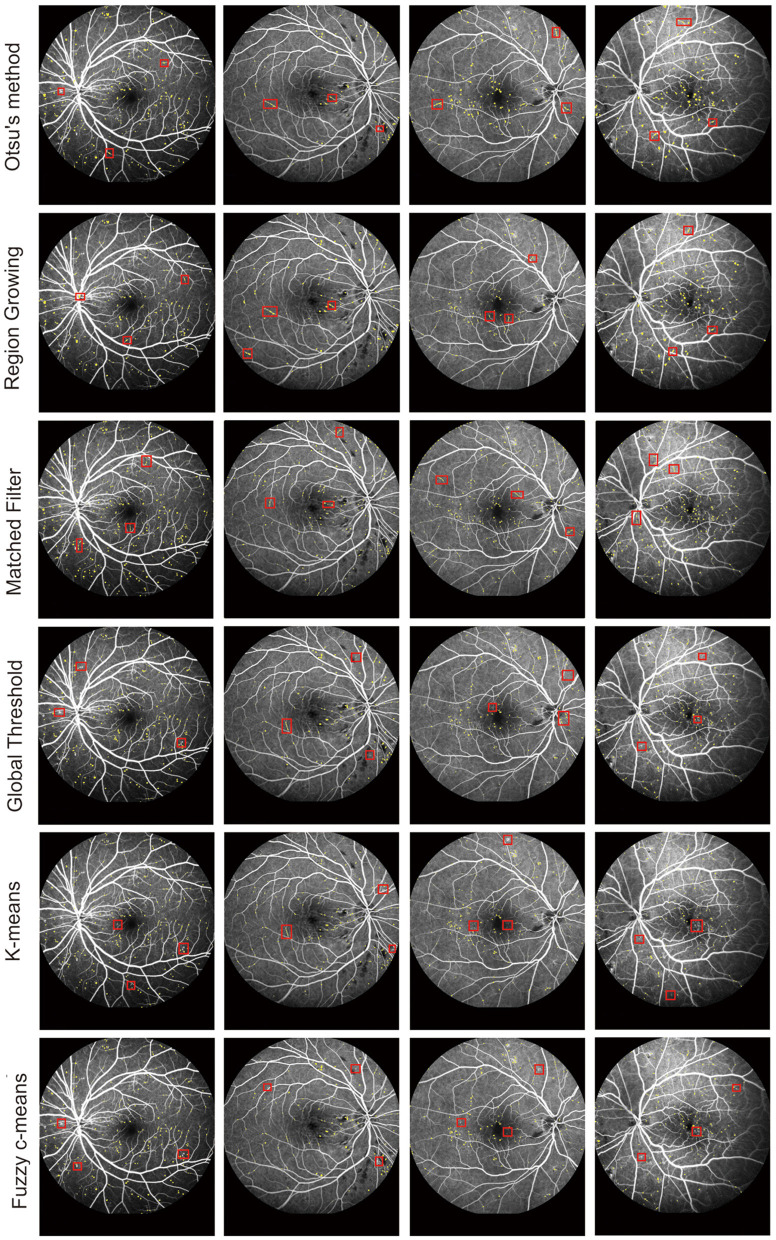
MA segmentation results from different models.

We further compared our proposed model against other MA detection models, such as Otsu's method (33), Region Growing (34), MF (25), Global Threshold (35), K-means, (36) and Fuzzy c-means, (37) to evaluate MA detection performance. The results of MA detection are shown in Figure 4, and the metrics of MA evaluation are shown in Table 2.

**Table 2:** Comparison of microaneurysm (MA) segmentation performance between our proposed model and other previously reported models.

**Model**	**Evaluation metrics**				
	**Acc (%)**	**Se (%)**	**Sp (%)**	**PPV (%)**	**IOU (%)**
Otsu's method	99.71 ± 0.08	79.55 ± 0.97	99.80 ± 0.08	63.85 ± 0.85	54.43 ± 1.11
Region growing	99.73 ± 0.07	81.80 ± 0.89	99.80 ± 0.07	63.92 ± 0.82	55.42 ± 1.07
Matched filter	99.73 ± 0.07	84.60 ± 0.57	99.79 ± 0.06	63.57 ± 0.80	57.40 ± 0.99
Global threshold	99.73 ± 0.08	78.06 ± 0.75	99.82 ± 0.06	62.88 ± 0.88	53.43 ± 1.12
*K*-means	99.76 ± 0.07	80.39 ± 0.68	99.83 ± 0.05	60.27 ± 0.75	52.36 ± 0.98
Fuzzy c-means	99.74 ± 0.06	76.52 ± 0.56	99.84 ± 0.05	63.23 ± 0.57	52.71 ± 0.92
Our model	99.80 ± 0.05	92.10 ± 0.20	99.85 ± 0.04	75.07 ± 0.44	70.57 ± 0.55

As shown in Figure 3 and Table 2, Otsu's method, Region Growing, MF, Global Threshold, K-means, and Fuzzy c-means models could not accurately detect the boundaries of MA regions and normal regions. Additionally, there were some omissions and false detections, which are marked by red squares in Figure 4. The proposed model had greater values of PPV and IOU than other models. Furthermore, our proposed model demonstrated performance in MA detection that closely aligned with the benchmark standard, surpassing the performance of other MA detection models.

To further evaluate MA detection performance, we used a publicly available dataset, which was obtained during a study conducted at the Persian Eye Clinic (Feiz Hospital) in Isfahan University of Medical Sciences (32), including retinal images from 70 patients, with 30 samples classified as normal and 40 samples representing different stages of DR. As shown in Table 3, MA detection using our proposed model had an average accuracy of 99.42%, a sensitivity of 90.21%, a specificity of 98.86%, a PPV of 71.93%, and an IOU of 64.89%, showing an obvious advantage over other MA detection models.

**Table 3:** Comparison of MA detection performance between our proposed model and previous detection models using the publicly available dataset.

**Model**	**Evaluation metrics**				
	**Acc (%)**	**Se (%)**	**Sp (%)**	**PPV (%)**	**IOU (%)**
Otsu's method	95.37 ± 0.12	79.76 ± 0.97	97.43 ± 0.16	64.43 ± 0.95	55.83 ± 1.05
Region growing	98.78 ± 0.11	82.22 ± 0.29	98.75 ± 0.21	67.21 ± 0.65	56.87 ± 1.21
Matched filter	98.54 ± 0.15	83.76 ± 0.29	98.46 ± 0.11	66.45 ± 0.86	56.87 ± 0.76
Global threshold	98.76 ± 0.09	79.12 ± 0.69	99.29 ± 0.16	64.64 ± 0.72	55.65 ± 1.08
*K*-means	98.55 ± 0.12	81.43 ± 0.87	99.12 ± 0.21	63.32 ± 0.86	54.65 ± 0.91
Fuzzy c-means	97.87 ± 0.11	79.47 ± 0.72	98.54 ± 0.13	64.54 ± 0.75	53.81 ± 0.87
Our model	99.42 ± 0.35	90.21 ± 0.54	98.86 ± 0.12	71.93 ± 0.41	64.89 ± 0.35

## 4 Discussion

MA detection is highly important for the diagnosis of DR (5). FFA is a technique used for the evaluation of retinal and choroidal circulation. MAs are immediately visible following the arterial phase of FFA (33). In this study, we propose a three-step model for MA detection in FFA images. Initially, FFA image pre-processing is conducted to enhance the contrasts of FFA images. Subsequently, candidate MA regions are coarsely segmented using an improved MF algorithm. Finally, real MA regions are identified through a morphological strategy. This proposed model aims to enhance the accuracy and efficiency of MA detection in FFA images, thus aiding in the early diagnosis and management of DR.

Automatic segmentation of MAs is still a tricky problem due to their tiny sizes, low contrasts, and high similarities to retinal vessels. The high-noise and low-contrast of FFA images can also affect the quality of FFA images and reduce the accuracy of MA detection (33). The goal of image enhancement is to decrease image noise and enhance the contrasts of the targets and backgrounds. In this study, top-hat transformation, gray-stretching, and a Gaussian filter were used for the improvement of FFA image quality. Top-hat transformation and gray-stretching can efficiently solve the problem of uneven illumination, while a Gaussian filter can efficiently reduce the potential impacts of retinal noises on FFA images.

We also evaluated the MA detection performance of the proposed model by comparing it with other MA detection methods. Compared with Otsu's method, Region Growing, MF, K-means, Global Threshold, and Fuzzy c-means (3, 25, 34–37), the proposed model has the greatest accuracy and efficiency for MA detection in FFA images. The evaluation metrics of the proposed model, including accuracy, sensitivity, specificity, PPV, and IOU, have the highest value. Moreover, the proposed model has a similar MA detection performance as the clinicians.

Recently, deep learning-based algorithms have gained popularity for medical image analysis. However, these algorithms typically demand high-performance computing resources, such as central processing units (CPUs) and graphics processing units (GPUs), as well as a substantial amount of labeled data for training. Unfortunately, many hospitals lack access to such resources and specialized personnel (38). Given this context, there is a pressing need for simpler methods for analyzing FFA images. In contrast to deep learning-based approaches, the proposed model does not necessitate a large number of labeled images or high-performance computing resources. Moreover, it offers comparable accuracy to manual labeling by clinicians but with faster detection speed. This feature makes it a practical and efficient solution for MA detection in clinical settings where resources and expertise may be limited.

## 5 Conclusion

This study provides a new model for the detection of MAs in FFA images, which consists of three steps. First, the quality of FFA images was improved by the image enhancement methods, including top-hat transformation, gray-stretching, and the Gaussian filter. Then, the candidate MAs were coarsely segmented by the MF algorithm. Finally, real MA regions were determined by the morphological strategy. Compared with manual MA labeling or other existing MA detection algorithms, the proposed model shows promising performance for the early diagnosis of DR by detecting MA lesions. This model is expected to assist ophthalmologists in efficiently detecting MA lesions, thereby enhancing the overall efficiency of DR diagnosis.

## 6 Limitations of this study

The number of MAs tends to increase as the severity of DR worsens. While the proposed model effectively detects the presence of MA lesions in FFA images, there are limitations to its clinical application. Indeed, MA formation is associated with various pathological changes such as basement membrane thickening, pericyte degeneration, and endothelial injury, which can lead to retinal vessel leakage, edema, and even hemorrhage. Given that vessel leakage, edema, and hemorrhage are closely linked to the size and volume of MAs, accurately detecting these parameters can provide additional valuable information for DR screening and monitoring. To achieve broader clinical applicability, the proposed model should be integrated with algorithms for detecting the size and volume of MAs, as well as for identifying edema and hemorrhages. This enhanced model would significantly improve the accuracy of assessing DR severity and estimating DR risk. Due to the high variability in pathological features and the quality of FFA images, deep learning techniques could play a crucial role in detecting and quantifying these features more accurately and efficiently. Therefore, in the future, we plan to incorporate deep learning approaches to further enhance the efficiency of MA detection in FFA images and improve the overall diagnostic capabilities for DR.

## Data availability statement

The original contributions presented in the study are included in the article/supplementary material, further inquiries can be directed to the corresponding authors.

## Ethics statement

Ethical review and approval was not required for the study on human participants in accordance with the local legislation and institutional requirements. Written informed consent from the (patients/participants OR patients/participants legal guardian/next of kin) was not required to participate in this study in accordance with the national legislation and the institutional requirements.

## Author contributions

QJ: Data curation, Writing – original draft, Writing – review & editing. JL: Conceptualization, Data curation, Writing – original draft. QM: Data curation, Formal analysis, Writing – original draft. MY: Conceptualization, Formal analysis, Writing – original draft. ZW: Conceptualization, Software, Writing – original draft. BY: Conceptualization, Writing – review & editing.
